# A novel hyperspectral compressive sensing framework of plant leaves based on multiple arbitrary-shape regions of interest

**DOI:** 10.7717/peerj-cs.802

**Published:** 2021-11-25

**Authors:** Yuewei Jia, Lingyun Xue, Ping Xu, Bin Luo, Ke-nan Chen, Lei Zhu, Yian Liu, Ming Yan

**Affiliations:** 1 College of Automation, Hangzhou Dianzi University, Hangzhou, Zhejiang Province, China; 2 Intelligent Equipment Research Center, Beijing Academy of Agriculture and Forestry Sciences, Beijing, China

**Keywords:** Plant leaf hyperspectral images, Regions of interest, Compressive sensing, Spectral index

## Abstract

Massive plant hyperspectral images (HSIs) result in huge storage space and put a heavy burden for the traditional data acquisition and compression technology. For plant leaf HSIs, useful plant information is located in multiple arbitrary-shape regions of interest (MAROIs), while the background usually does not contain useful information, which wastes a lot of storage resources. In this paper, a novel hyperspectral compressive sensing framework for plant leaves with MAROIs (HCSMAROI) is proposed to alleviate these problems. HCSMAROI only compresses and reconstructs MAROIs by discarding the background to achieve good reconstructed performance. But for different plant leaf HSIs, HCSMAROI has the potential to be applied in other HSIs. Firstly, spatial spectral decorrelation criterion (SSDC) is used to obtain the optimal band of plant leaf HSIs; Secondly, different leaf regions and background are distinguished by the mask image of the optimal band; Finally, in order to improve the compression efficiency, after discarding the background region the compressed sensing technology based on blocking and expansion is used to compress and reconstruct the MAROIs of plant leaves one by one. Experimental results of soybean leaves and tea leaves show that HCSMAROI can achieve 3.08 and 5.05 dB higher PSNR than those of blocking compressive sensing (BCS) at the sampling rate of 5%, respectively. The reconstructed spectra of HCSMAROI are especially closer to the original ones than that of BCS. Therefore, HCSMAROI can achieve significantly higher reconstructed performance than that of BCS. Moreover, HCSMAROI can provide a flexible way to compress and reconstruct different MAROIs with different sampling rates, while achieving good reconstruction performance in the spatial and spectral domains.

## Introduction

Hyperspectral imaging (HSI) provides a potential insight into plant-pathogen interactions (Mahlein et al., 2018). Physiological and biochemical parameters of plant leaves can be effectively predicted with the reflectance spectra extracted from plant leaf hyperspectral images (HSIs), which can be an appropriate measure to evaluate the health and vitality state of plants. HSI has been used to do early detection of grapevine leafroll disease in a red-berried wine grape cultivar (Gao et al., 2020). The moisture content of tea leaves can be detected with hyperspectral imaging technology (Wei et al., 2019). HSIs can detect leaves at the stages of freezing and post-thawing injury, and quantify the impacts of freezing injury on leaf water and pigment contents (Wei et al., 2017). Therefore, with the wide use of HSIs in plant leaves, it brings huge challenge to their acquisition, storage and transmission.

Compressive sensing (CS) can not only combine the process of acquisition and compression, but also reconstruct them accurately. In recent years, CS has been a hotspot in the field of HSI. The applications of CS includes reconstruction, denoising, and unmixing et al. For reconstruction applications, based on a multidimensional multiplexing (MDMP) CS scheme, a tensor nonlinear CS (T-NCS) algorithm for noniterative recovery of HSIs using example-aided and self-learning compressive hyperspectral imaging (CHI) approaches was proposed (Yang et al., 2015). Martin, Bioucas-Dias & Plaza (2015) exploited the high correlation existing among the components of the hyperspectral data sets to introduce a new hyperspectral coded aperture (HYCA), which largely reduced the number of measurements to reconstruct the original data correctly. Zhang et al. (2016b) brought forward a structured sparsity-based hyperspectral blind CS (SSHBCS) algorithm in which a unified optimization framework was derived to jointly learn the dictionary and the corresponding sparse representation from the measurements. Wang et al. (2017) considered a single HSI as a tensor with three modes (width, height and band) and then identified the hidden spatial-spectral structures using low-rank Tucker decomposition to model the global spatial-spectral correlation. Gelvez, Rueda & Arguello (2017) proposed a joint sparse and low rank HSI recovery algorithm, which included a nuclear norm regularization term in the objective function, and then obtains the optimal solution through iteration. Xu et al. (2018) proposed a Mahalanobis distance-regularized tensor robust principal component analysis framework for hyperspectral CS with anomaly detection which reconstructed the HSI and detected the anomalies simultaneously. Meza et al. (2018) studied the spatial and spectral correlation of HSI cube, and reconstructed hyperspectral data through non-local means regularization and Bregman optimization techniques. Xue et al. (2019) proposed a nonlocal tensor sparse and low-rank regularization (NTSRLR) approach, which can encode essential structured sparsity of an HSI and explore its advantages for HSI-CSR task. For denoising applications, Hassanzadeh & Karami (2017) put forward a new lossy compression and noise reduction algorithm for HSIs based on non-negative Tucker decomposition (NTD) and CS. To model the distribution of structured sparsity in HSIs, and to make it adaptable to the unknown noise, Zhang et al. (2016a) proposed a novel reweighted Laplace prior based on hyperspectral compressive sensing (RLPHCS) algorithm. Tan et al. (2016) extended the AMP-Wiener algorithm to three-dimensions and applied it to the coded aperture snapshot spectral imager (CASSI) system for hyperspectral image reconstruction. For unmixing applications, Li et al. (2012) proposed a novel numerical procedure, and proposed an augmented Lagrangian algorithm for reconstructing the model. Junmin & Jiangshe (2014) casted the spectral unmixing into the CS framework by multiplying a random Gaussian measurement matrix on the SR model and investigated the effect of the proposed model on the recovered abundances obtained by the *l*1-minimization algorithms. A locally similar sparsity-based hyperspectral unmixing CS (LSSHUCS) method was proposed to unmix the HSIs with an established redundant endmember library (Zhang et al., 2016c). Based on spectral linear mixing model, a CS reconstruction algorithm with spectral unmixing for HSIs is proposed (Wang et al., 2018).

In terms of plant HSIs, especial for plant leaf HSIs, it is common that plant leaves belong to multiple arbitrary-shape regions of interest (ROIs) and background wastes too much memory resource. These above state-of-the-art hyperspectral CS algorithms can only compress and reconstruct the whole cubic HSIs and cannot process the arbitrary shape ROIs to achieve good compression performance.

In our previous works, we introduced the CS into the compression and reconstruction of plant HSIs and chose three critical physiological and biochemical parameters of plant to verify the retrieving efficacy of the proposed algorithms (Ping et al., 2016; Xu et al., 2017). In this paper, a general hyperspectral CS framework for plant leaf based on multiple arbitrary-shape ROIs (HCSMAROI) is proposed to further improve the compression efficiency of plant leaves HSIs at low sampling rates. HCSMAROI can not only compress different plant leaf HSIs, but also have the potential to be applied in other hyperspectral images with multiple arbitrary-shape bands of interest (MAROIs). HCSMAROI can compress different ROIs with different target rates. The spatial spectral decorrelation criterion (SSDC) is used to obtain the optimal band of plant leaf HSIs. Then, the labeled mask of the multiple arbitrary-shaped ROIs can be extracted according to the optimal band. After discarding the useless background data, each arbitrary-shape ROI can be divided into different blocks, which can be transformed into one-dimension data, and then compressed and reconstructed at the preset target bitrates using the random Gaussian matrix and staged orthogonal matching pursuit (StOMP) (Donoho et al., 2012).

The main contributions of this paper are as follows:

1. A novel hyperspectral CS framework is proposed to compress and reconstruct plant leaf HSIs;
2. The hyperspectral CS framework can not only be used in different plant leaf HSIs, but also have the potential to be applied in other HSIs;
3. A labeled mask is extracted to obtain multiple arbitrary-shape ROIs according to the optimal band;
4. Different arbitrary-shape ROIs can be compressed and reconstructed with different target bitrates after blocking and expansion.

## Algorithm

### HCSMAROI

**Figure 1:**
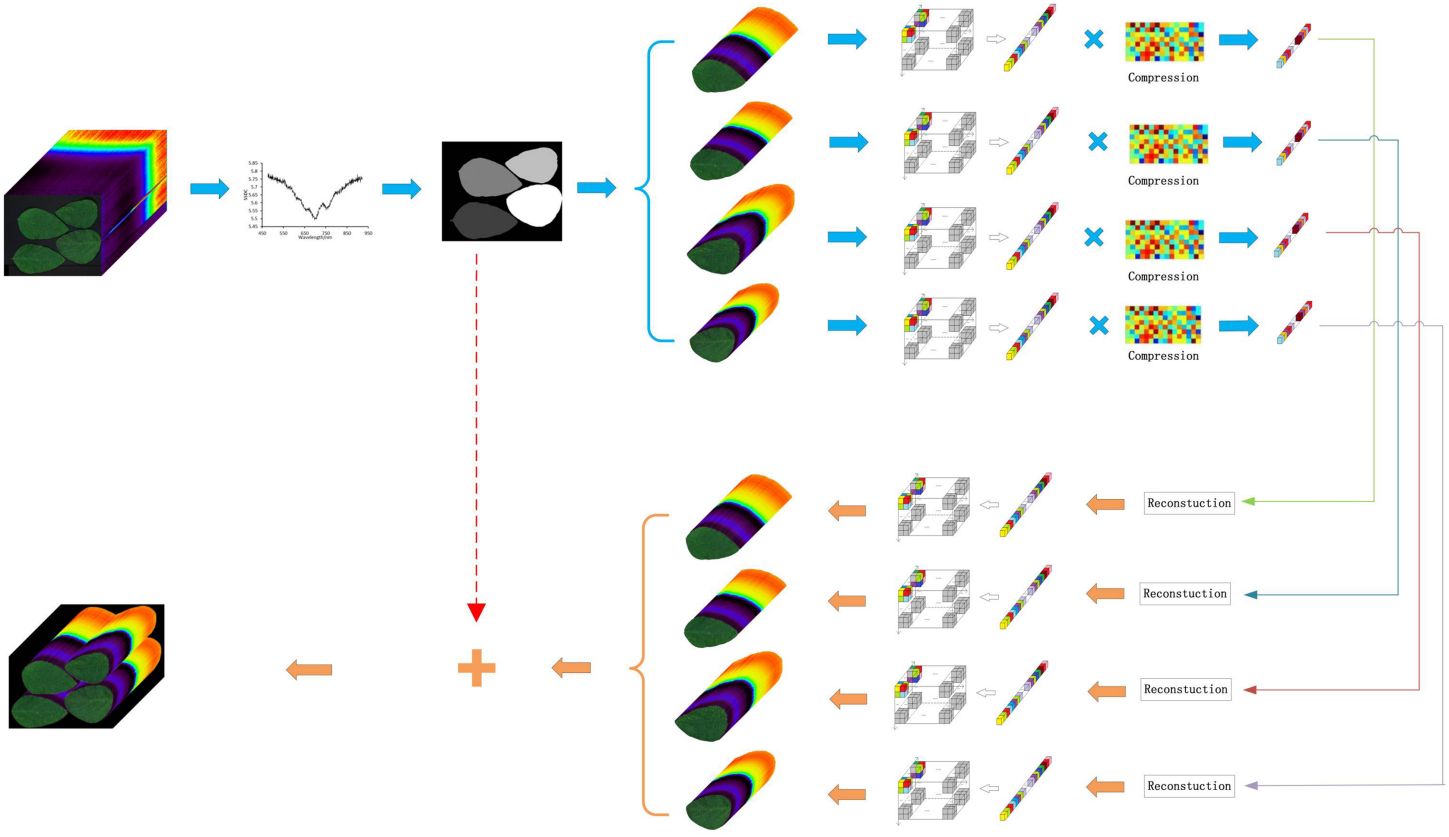
Overview of HCSMAROI.

For most hyperspectral applications, plant leaf HSIs can be divided into multiple arbitrary shape region of interests and a background region in the spatial domain. This framework has the novelty that it can compress different arbitrary shape ROIs independently with different target rates and discard the background region. It can not only compression plant leaf HSIs, but also have the potential to be applied in other hyperspectral images with MAROIs. Fig. 1 shows the overview of HCSMAROI.

Different types of HSIs can be effectively divided with appropriate strategies. For plant leaf HSIs, SSDC can be used to obtain the optimal band and then different ROIs can be labeled. For other types of HSIs, such as HSIs of satellite remote sensing, many image terrain classification algorithms can be used to extract the ROIs.

The proposed framework can be summarized as follows:

Firstly, SSDC is used to obtain the key band of plant leaf HSIs; Secondly, the primary mask of the multiple arbitrary-shape ROIs could be extracted, and the final labeled mask of different ROIs can also be obtained in the key band; Thirdly, after discarding the useless background data for saving the memory storage, all arbitrary-shape ROIs of plant HSIs can be divided into blocks in the spatial domain and transformed into one-dimensional data one by one, then a random Gaussian matrix is selected to compress each arbitrary-shape ROI; Fourthly, StOMP is chosen to reconstruct each arbitrary-shape ROI; Finally, the reconstructed plant leaf HSI can be obtained by combining all reconstructed MAROIs.

### Extraction the optimal band using SSDC

Gao et al. (2013) had compared the noise estimation performance of six main evaluation methods of homogeneous area (HA), geo-statistical (GS), local means and local standard deviations (LMLSD), SSDC, residual-scaled local standard deviation (RLSD), and homogeneous regions division and spectral de-correlation (HRDSDC), and obtained the conclusion that SSDC was the most reliable evaluation method for hyperspectral noise estimation performance among these six evaluation methods. Therefore, SSDC is chosen to evaluate the image quality of HSIs. After calculating the SSDC values of all bands, the band with the minimal SSDC is regarded as the optimal band.

### ROI mask extraction and labeling

After obtaining the optimal band, *k*-means clustering algorithm is used to extract the mask for the arbitrary-shape ROIs of the HSIs. According to the characteristics of the optimal band, the number of classes, *m*, needs to be preset. The optimal band can be divided into *m* classes. But for the background region, the remaining *m*-1 classes can be combined to obtain the primary binary mask and construct the whole leaf regions.

Each region in the primary binary mask can represent a ROI in the optimal band. Each 8-connected region in the primary binary mask can be labeled with an index value to construct the labeled mask.

### Blocking and expansion for arbitrary-shape ROIs

In order to obtain good reconstructed performance, hyperspectral data of ROIs were compressed and reconstructed while those of background were discarded. Because of stronger spectral correlation of HSIs, compared with the spatial correlation, all ROIs were divided into multiple sub blocks only in the spatial domain. In the light of our extensive experiments, spatial block size of 2 × 2 can be used to achieve good reconstructed performance.

**Figure 2:**
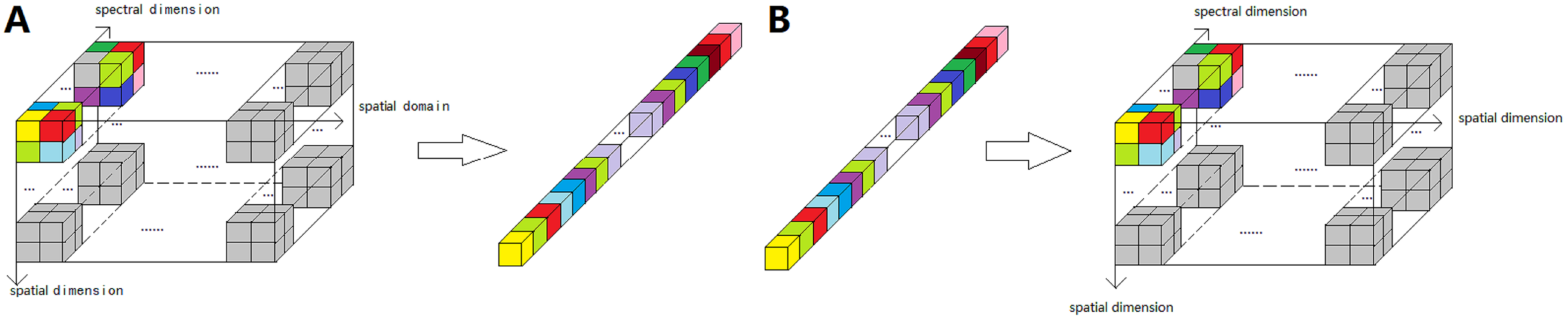
The expansion and inverse expansion process scheme of a block. (A) Expansion process. (B) Inverse expansion process.

Considering the high correlation of neighboring pixels in the spatial domain, each block of the same position of all bands is firstly expanded into one-dimensional data in the order of zigzag band by band, and then combined to construct the whole one-dimensional data. Figure 2 shows the expansion and inverse expansion scheme of a block.

### CS and reconstruction for arbitrary-shape ROIs

The measurement matrix ${\phi} \in {R^{M \times N}}$ is used to sample each one-dimension expansion data of arbitrary-shape ROIs randomly; *M* is the signal length after sampling, *N* is the expansion length of a block, *M << N*; $\phi$ is the random Gauss measurement matrix.

The sampling rate can be defined as follow.

(1) $$R = {M \over N}$$

The sampling process is as below.

(2) $$Y = \Phi X$$

where *X* is a one-dimension expansion data, and *Y* is the observed data. We use Discrete Cosine Transform (DCT) matrix to construct the sparse basis $\psi$, $\psi \in {R^{N \times N}}$, ${\psi ^T} \times \psi = I$, $I$ is the identity matrix, which is an orthonormal matrix. *Ψ* is given by

(3) $$\Psi = {1 \over {\sqrt N }}\left[ {\matrix{{\vskip0.5pc} 1  1  \ldots  1 \cr {\vskip1pc}{\sqrt 2 \cos \displaystyle{\pi \over {2N}}}  {\sqrt 2 \cos \displaystyle{{3\pi } \over {2N}}}  \ldots  {\sqrt 2 \cos \displaystyle{{\left( {2N - 1} \right)\pi } \over {2N}}} \cr {\vskip1pc}\ldots  \ldots  \ldots  \ldots \cr {\sqrt 2 \cos \displaystyle{{\left( {N - 1} \right)\pi } \over {2N}}}  {\sqrt 2 \cos \displaystyle{{3\left( {N - 1} \right)\pi } \over {2N}}}  \ldots  {\sqrt 2 \cos \displaystyle{{\left( {2N - 1} \right)\left( {N - 1} \right)\pi } \over {2N}}} \cr } } \right]$$

The sensing matrix *A* is constructed by *Ψ* and *Φ* and is shown by Eq. (4). To ensure the convergence of the reconstruction algorithm, the sensing matrix *A* must satisfy the principle of the restricted isometry property (RIP) (Candès, 2008).

(4) $$A = \Phi \Psi$$

Due to the high efficiency and high accuracy of StOMP, it is chosen as the reconstructed algorithm to reconstruct each one-dimension expansion data.

The inverse expansion process (Fig. 2B) is performed to reconstruct a block. After all blocks have been reconstructed, each ROI of plant leaf HSIs can be reconstructed. Finally, the reconstructed plant leaf HSI can be obtained by combining all reconstructed MAROIs according to the labeled mask.

## Performance evaluation methods

Peak Signal to Noise Rate (PSNR) is chosen to evaluate the reconstructed performance of each ROI in the spatial domain. Mean Square Error (MSE) and PSNR of ROI are defined as follows:

(5) $$MS{E_{ROI\left( {idx} \right)}} = {1 \over {Num\left( {idx} \right)}}\sum\limits_{\left( {i,j,k} \right) \in ROI\left( {idx} \right)} {{{\left| {Xrec\left( {i,j,k} \right) - Xori\left( {i,j,k} \right)} \right|}^2}}$$

(6) $$PSN{R_{ROI\left( {idx} \right)}} = 10 \times {\log _{10}}\left( {{{{{\left( {{2^n} - 1} \right)}^2}} \over {MS{E_{ROI\left( {idx} \right)}}}}} \right)$$

where *idx* is the index of ROIs, $Are{a_{ROI(idx)}}$ is the total pixel count of the *idx*-*th* ROI, *Xrec* is the reconstructed data, *Xori* is the original data, and *n* is the number of bits per sample.

The reconstructed plant spectral indices Triangular Vegetation Index (TVI) and Double Difference (DD) are used to evaluate the reconstructed effectiveness in the spectral domain. Two physiological indices in Table 1 are chosen to evaluate performance, where *Rx* represents the spectral reflectance at the wavelength of *x* nm (Broge & Leblanc, 2000; le Maire, François & Dufrêne, 2004).

**Table 1:** Spectral indices of TVI and DD.

Spectral index	Definition
TVI	0.5*[120*(R750-R550)-200*(R670-R550)]
DD	(R750-R720)-(R700-R670)

## Experimental results and analysis

In the experiments, two types of plant leaf HSIs are chosen. One is soybean leaf and the other is tea leaf.

### HSIs of soybean leaves and tea leaves

In this study, a hyperspectral imaging system ImSpector V10E (ImSpector V10E, SPECIM, Finland) covering the spectral wavelengths of 400–1,000 nm was used to snap the soybean leaf HSIs. The system includes a CCD camera (DL-604M, Andor, Ireland), an imaging spectrograph, a lens, a light sources provided by 150 W quartz tungsten halogen lamp (2900-ER, Illumination, Santa Monica, CA, USA) and software (Isuzu Optics Corp, Zhubei, Taiwan) for the computer operating the spectral image system. The spectral resolution is 0.65 nm and the resolution of CCD array detector of the camera is 1,344 × 1,024. The system is driven by electronically controlled displacement platform (IRCP0076, Isuzu Optics Corp, Taiwan) to scan the samples line by line.

**Figure 3:**
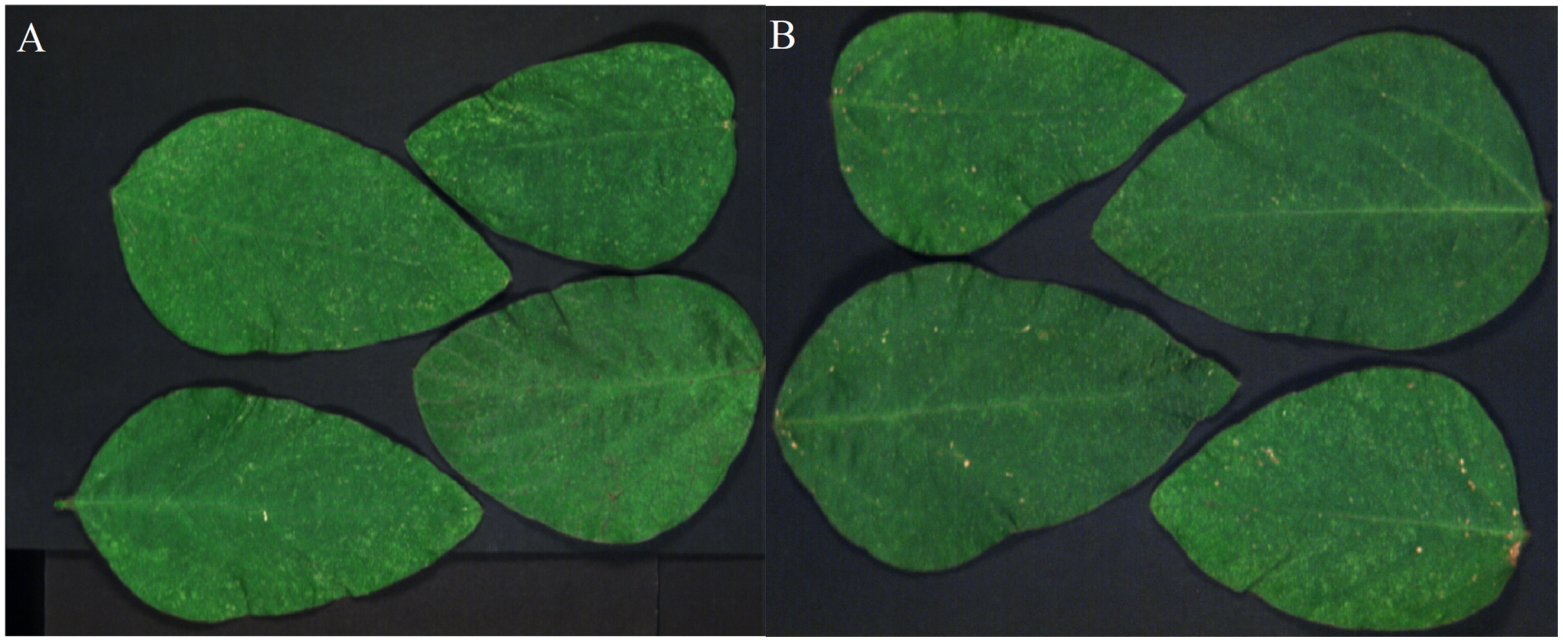
RGB images for raw soybean leaf HSIs. (A) The first soybean leaf HSIs. (B) The second soybean leaf HSIs.

Two sets of soybean leaf HSIs are used in the experiment. The wavelength range of the first soybean leaf HSIs is from 476 nm to 939 nm, in which there are totally 596 bands. A single pixel is defined by a 16-bit unsigned integer and their resolution is 834 × 1,004, that is to say, the total pixel count of a band is 837,336. The wavelength range of the second soybean leaf HSIs is from 460 to 900 nm, in which there are totally 568 bands, and their resolution is 706 × 887. The bands of 666, 558 and 476 nm of these two sets of soybean leaf HSIs are selected as the red, green and blue channels to construct the false color composite RGB images as shown in Figs. 3A and 3B, respectively.

**Figure 4:**
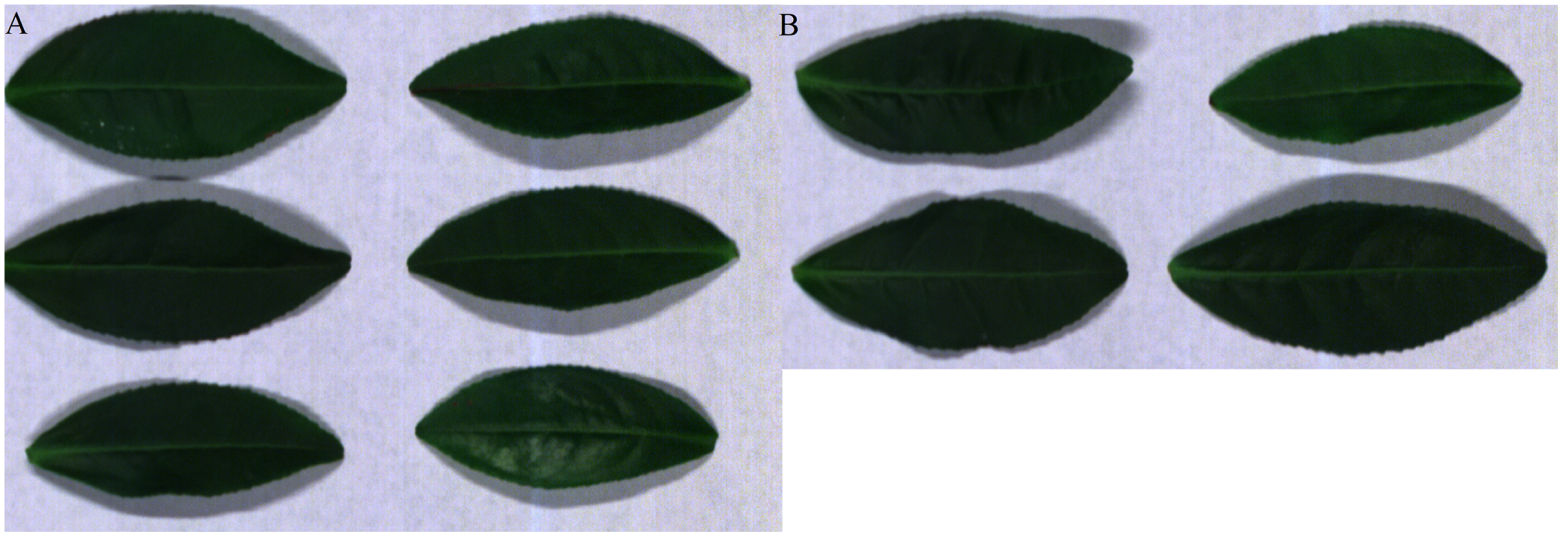
RGB images for raw tea leaf HSIs. (A) The first tea leaf HSIs. (B) The second tea leaf HSIs.

The tea leaf HSIs in our previous research (Xu et al., 2017) are used in this paper. A single pixel is defined by a 12-bit unsigned integer. The resolution of the first tea leaf HSIs is 435 × 642, in which there are totally 381 bands. The wavelength range of the second tea leaf HSIs is from 449 to 915 nm, in which there are totally 369 bands, and their resolution is 294 × 627. The image of 666, 555 and 454 nm of tea leaf HSIs are selected as the red, green and blue channels of the false color composite RGB images as shown in Figs. 4A and 4B.

### Mask extraction

#### Primary mask extraction

**Figure 5:**
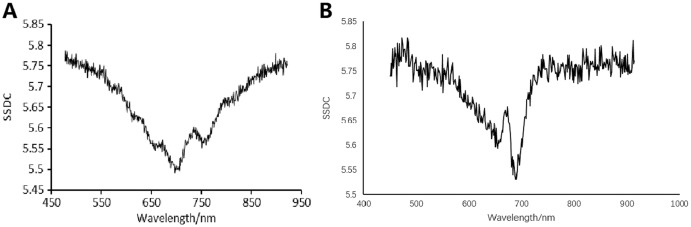
SSDC wavelength curves. (A) SSDC wavelength curve for the first soybean leaf HSIs. (B) SSDC wavelength curve for the first tea leaf HSIs.

**Figure 6:**
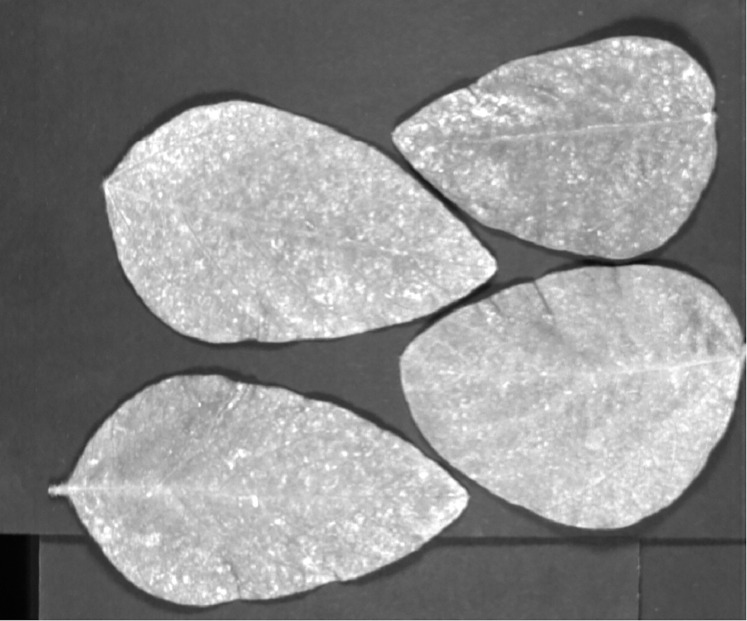
Gray image of the 702 nm band for the first soybean leaf HSIs.

**Figure 7:**
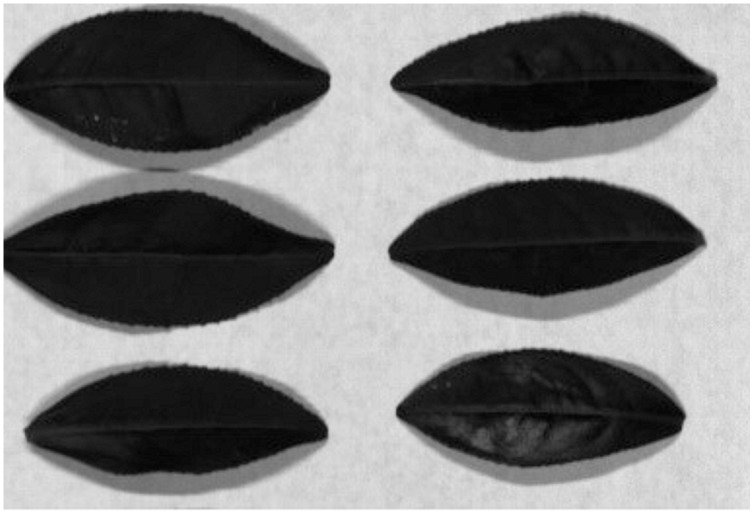
Gray image of the 690 nm band for the first tea leaf HSIs.

Figure 5A indicates the SSDC curve of the first soybean leaf HSIs in which the 702 nm band shows the lowest SSDC value. Figure 5B shows the SSDC curve of the first tea leaf HSIs in which the 690 nm band obtains the lowest SSDC value. Therefore, the 702 nm band and the 690 nm band are chosen as the optimal bands which can be used to extract the mask of multiple arbitrary-shape ROIs of four pieces of soybean leaves and six pieces of tea leaves, respectively. Figures 6 and 7 show the 702 nm band image of soybean leaf HSIs and the 690 nm band image of tea leaf HSIs, respectively.

**Figure 8:**
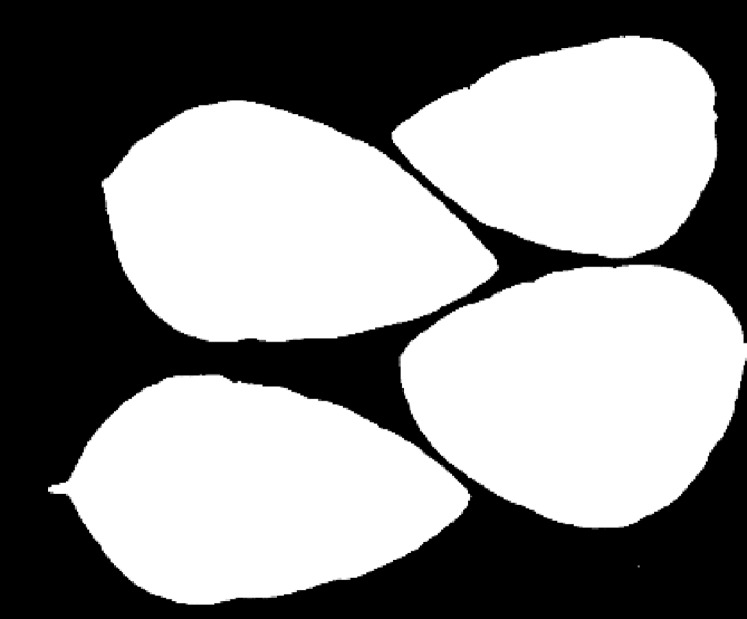
The primary binary mask for the first soybean leaf HSIs.

**Figure 9:**
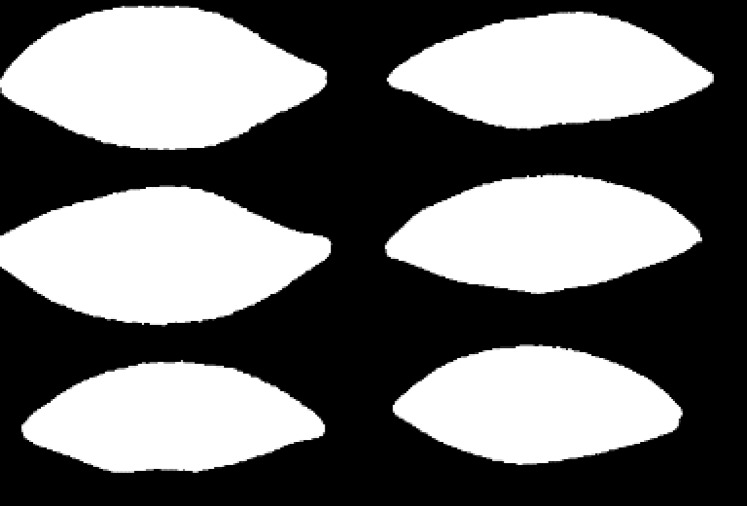
The primary binary mask for the first tea leaf HSIs.

The *k*-means clustering algorithm is used to cluster these optimal bands. The number of classifications *m* is set as four and three for the first soybean leaf HSIs and the first tea leaf HSIs, respectively. Figures 8 and 9 show their primary binary masks by the *k*-means clustering algorithm.

#### Labeled mask extraction

The ROI regions of the primary mask are labeled from left to right and from top to bottom. In the labeled mask, the larger ROI label value, the higher its gray value.

**Figure 10:**
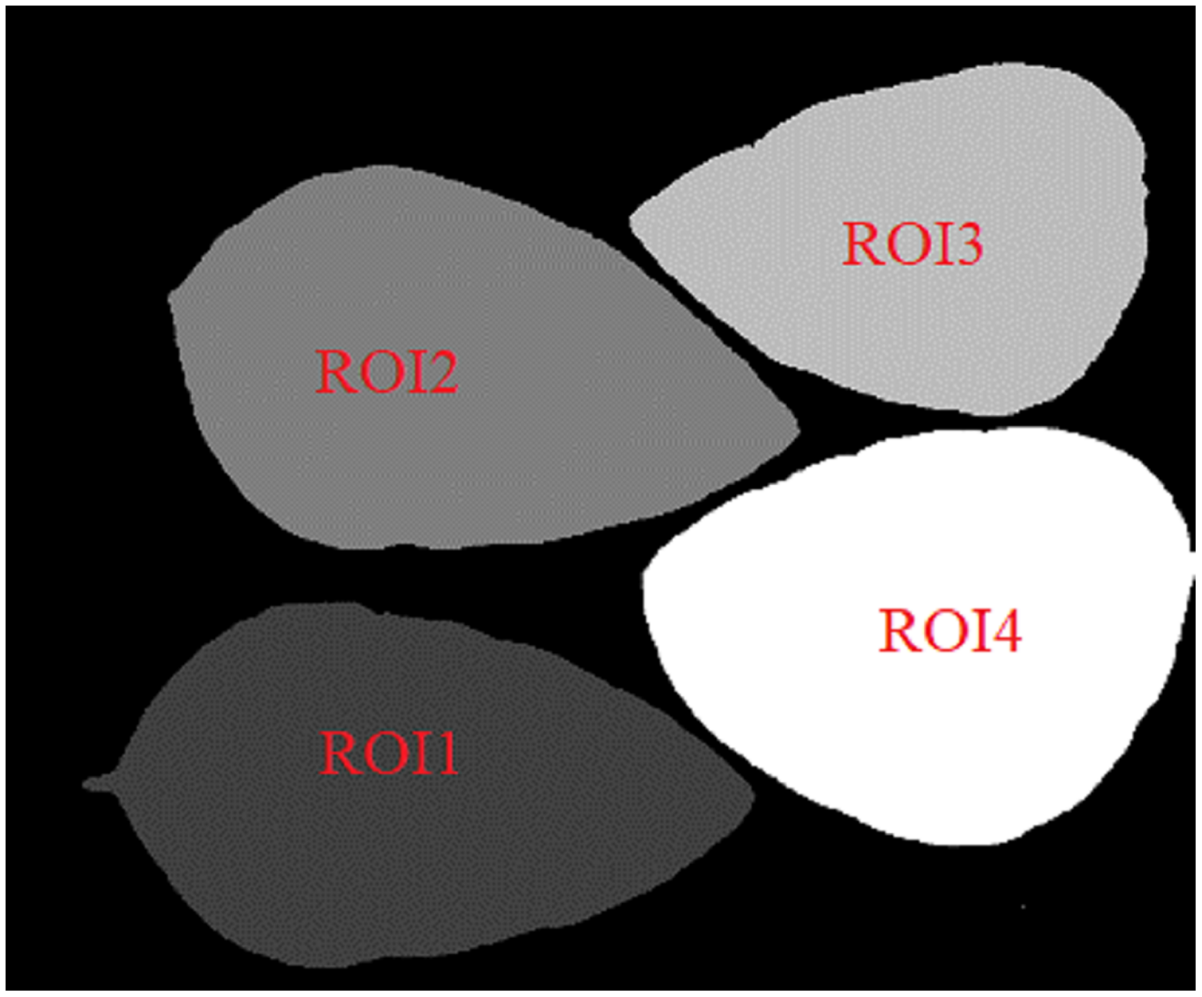
The labeled mask for the first soybean leaf HSIs.

**Figure 11:**
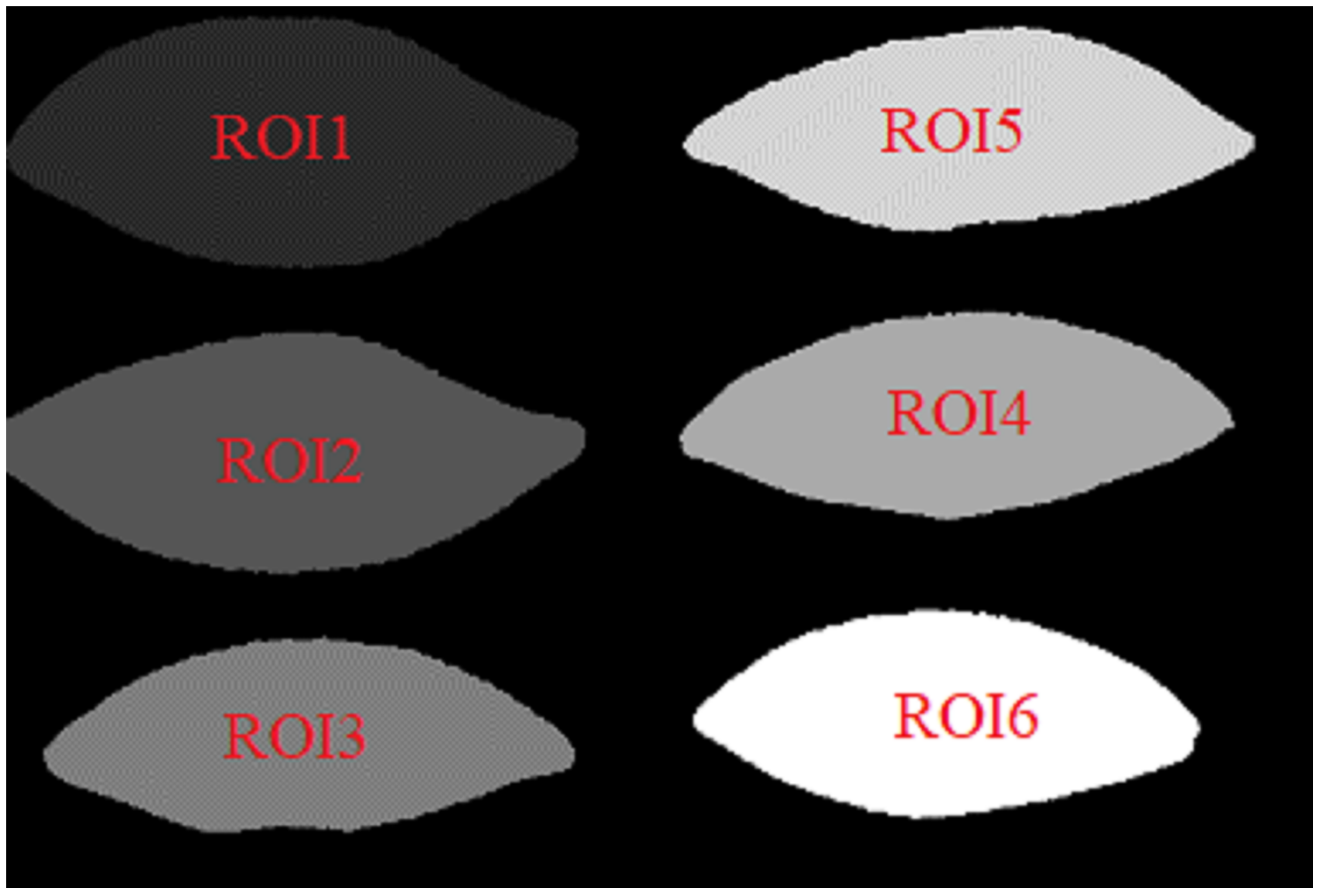
The labeled mask for the first tea leaf HSIs.

Figures 10 and 11 show the labeled masks of multiple arbitrary-shape ROIs of soybean leaves and tea leaves. The total pixel area *Area_ROIs* of all ROIs in the labeled mask can be obtained as follows.

(7) $$Are{a_{ROIs}} = \sum\limits_{{\rm{i}} = 1}^{\rm{n}} A re{a_{ROI({\rm{i}})}}$$

where $Are{a_{BOI({\rm{i}})}}$ is pixel area of the *ith* ROI in the labeled mask, *n* is the total number of ROIs.

### Reconstruction performance analysis of multiple arbitrary-shape ROIs using the primary mask

In this case, multiple arbitrary-shape ROIs can be compressed and reconstructed using the primary binary mask at the whole target sampling rate. Single spectral compressive sensing (SSCS) and blocking compressive sensing (BCS) are chosen to make the performance comparison. The sampling rates of soybean leaf HSIs are 5%, 10%, 15% and 20%, and the sampling rates of tea leaf HSIs are 3%, 5%, 8%, 12%, 17% and 20%. That is to say, the actual sampling rates of different ROIs are respectively written as follows.

(8) $$Rati{o_p} = {{Are{a_{whole}}} \over {Are{a_{ROIs}}}} \times p$$

where $Are{a_{whole}}$ is the whole area of all ROIs in the primary mask, $p$ is the sampling rates of the HSIs, and $Rati{o_p}$ is the sampling rates of all ROIs.

#### Spatial domain reconstruction analysis

**Figure 12:**
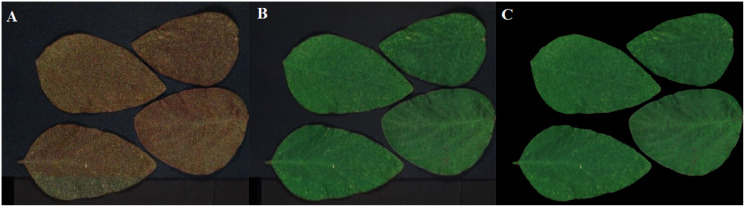
The reconstructed images for the first soybean leaf HSIs at the sampling rate of 5%. (A) SSCS. (B) BCS. (C) HCSMAROI.

**Figure 13:**
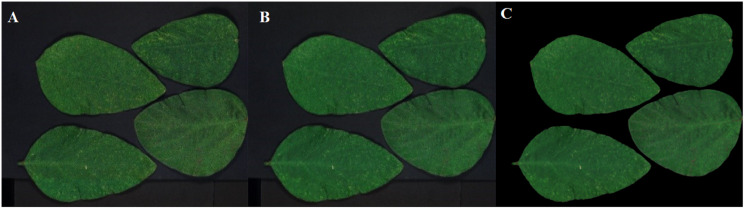
The reconstructed images for the first soybean leaf HSIs at the sampling rate of 10%. (A) SSCS. (B) BCS. (C) HCSMAROI.

**Figure 14:**
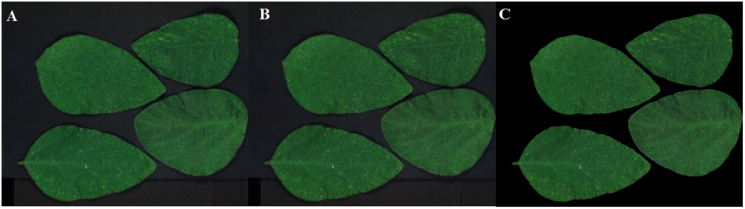
The reconstructed images for the first soybean leaf HSIs at the sampling rate of 15%. (A) SSCS. (B) BCS. (C) HCSMAROI.

**Figure 15:**
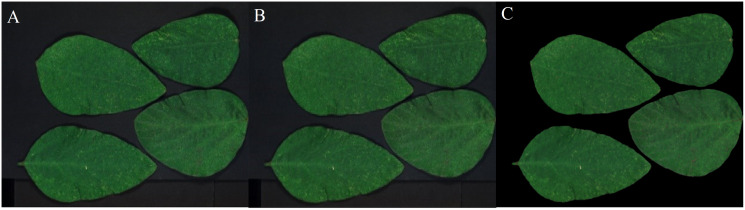
The reconstructed images for the first soybean leaf HSIs at the sampling rate of 20%. (A) SSCS. (B) BCS. (C) HCSMAROI.

**Figure 16:**
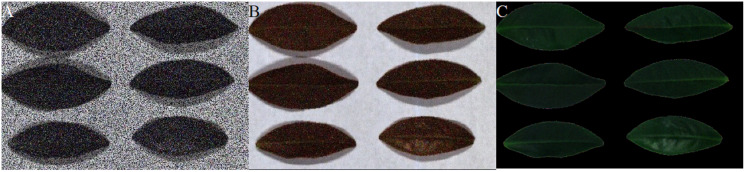
The reconstructed images for the first tea leaf HSIs at the sampling rate of 3%. (A) SSCS. (B) BCS. (C) HCSMAROI.

**Figure 17:**
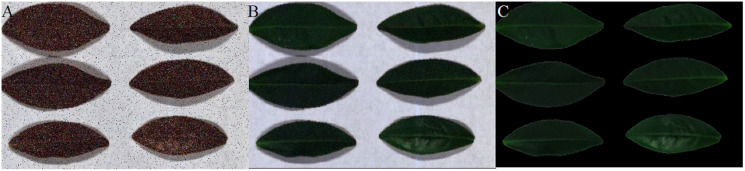
The reconstructed images for the first tea leaf HSIs at the sampling rate of 5%. (A) SSCS. (B) BCS. (C) HCSMAROI.

**Figure 18:**
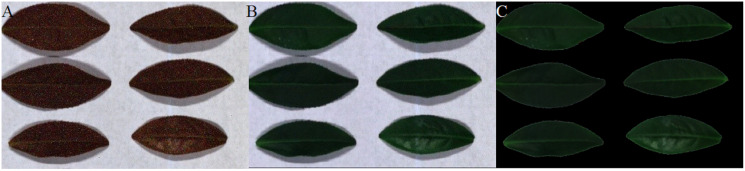
The reconstructed images for the first tea leaf HSIs at the sampling rate of 8%. (A) SSCS. (B) BCS. (C) HCSMAROI.

**Figure 19:**
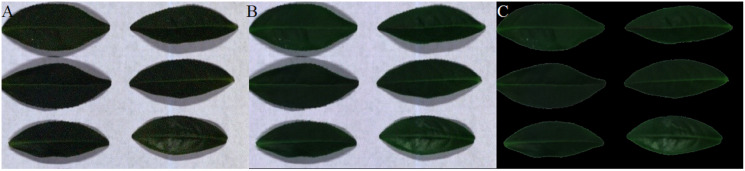
The reconstructed images for the first tea leaf HSIs at the sampling rate of 12%. (A) SSCS. (B) BCS. (C) HCSMAROI.

**Figure 20:**
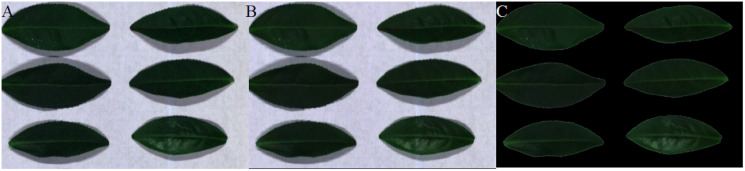
The reconstructed images for the first tea leaf HSIs at the sampling rate of 17%. (A) SSCS. (B) BCS. (C) HCSMAROI.

**Figure 21:**
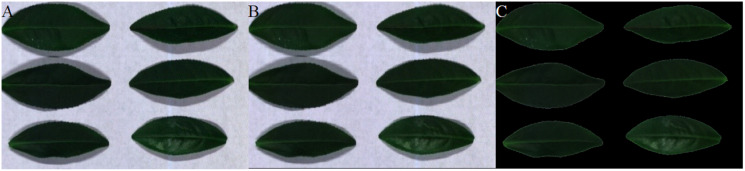
The reconstructed images for the first tea leaf HSIs at the sampling rate of 20%. (A) SSCS. (B) BCS. (C) HCSMAROI.

Figures 12–15 illustrate the experimental results of soybean leaf HSIs for SSCS, BCS and HCSMAROI at the sampling rates from 5% to 20%. Figures 16–21 illustrate the experimental results of tea leaf HSIs for SSCS, BCS and HCSMAROI at the sampling rates from 3% to 20%. Compared with SSCS, BCS preserves the spatial correlation to achieve better reconstructed image. HCSMAROI allocates all the bit rates to the leaf regions and can achieve significantly better reconstructed subjective quality for leaf regions than that of the other two algorithms at low sampling rates.

The reconstructed PSNRs performances of soybean leaf HSIs as shown in Table 2, the reconstructed PSNRs performances of tea leaf HSIs as shown in Table 3. The reconstructed PSNRs of HCSMAROI and BCS are higher than that of SSCS at different sampling rates. The PSNRs of reconstructed images of different algorithms are all improved with the increasing of the sampling rate. HCSMAROI achieves the highest PSNRs at all sampling rates. When the sampling rate is lower than 10%, the reconstructed PSNR values of HCSMAROI are significantly higher than that of BCS. When the sampling rate is greater than or equal to 20%, the reconstructed PSNR of HCSMAROI is close to that of BCS. It is shown that HCSMAROI can achieve quite good reconstructed PSNR in the spatial domain, especially at low sampling rate.

**Table 2:** Comparisons of reconstructed PSNRs/dB for soybean leaf HSIs for different algorithms.

Algorithms		Sampling rates			
		5%	10%	15%	20%
The first soybean leaf HSIs	SSCS	21.59 dB	31.70 dB	36.79 dB	38.51 dB
	BCS	33.25 dB	36.70 dB	38.19 dB	38.96 dB
	HCSMAROI	36.33 dB	38.88 dB	39.77 dB	40.24 dB
The second soybean leaf HSIs	SSCS	17.68 dB	30.91 dB	38.84 dB	40.60 dB
	BCS	34.40 dB	38.30 dB	39.78 dB	40.57 dB
	HCSMAROI	37.01 dB	39.80 dB	40.92 dB	41.48 dB

**Table 3:** Comparisons of reconstructed PSNRs/dB for tea leaf HSIs for different algorithms.

Algorithms		Sampling rates					
		3%	5%	8%	12%	17%	20%
The first tea leaf HSIs	SSCS	7.78 dB	10.06 dB	16.35 dB	24.55 dB	33.12 dB	35.03 dB
	BCS	17.83 dB	27.56 dB	32.45 dB	34.24 dB	35.30 dB	35.68 dB
	HCSMAROI	30.74 dB	33.61 dB	35.35 dB	36.39 dB	37.01 dB	37.26 dB
The second tea leaf HSIs	SSCS	8.19 dB	9.86 dB	16.14 dB	25.76 dB	32.66 dB	34.60 dB
	BCS	18.37 dB	27.61 dB	32.53 dB	34.44 dB	35.55 dB	35.95 dB
	HCSMAROI	29.73 dB	33.30 dB	35.30 dB	36.47 dB	37.17 dB	37.43 dB

#### Spectral domain reconstruction results analysis

**Figure 22:**
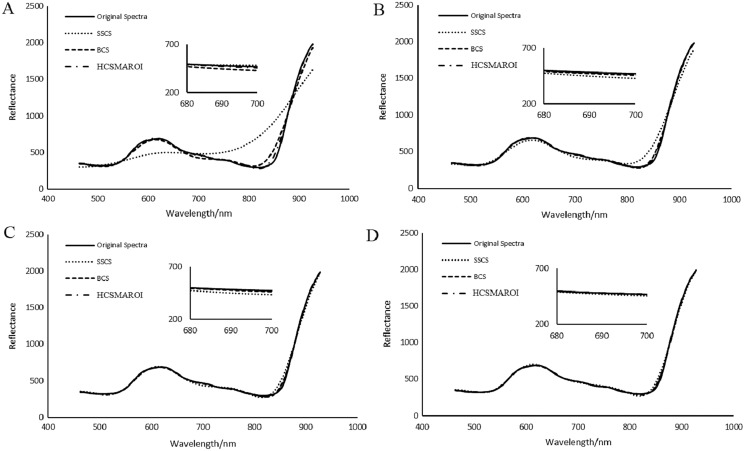
Comparisons of reconstructed spectra for the first soybean leaf HSIs. (A) The whole sampling rate of 5%. (B) The whole sampling rate of 10%. (C) The whole sampling rate of 15%. (D) The whole sampling rate of 20%.

**Figure 23:**
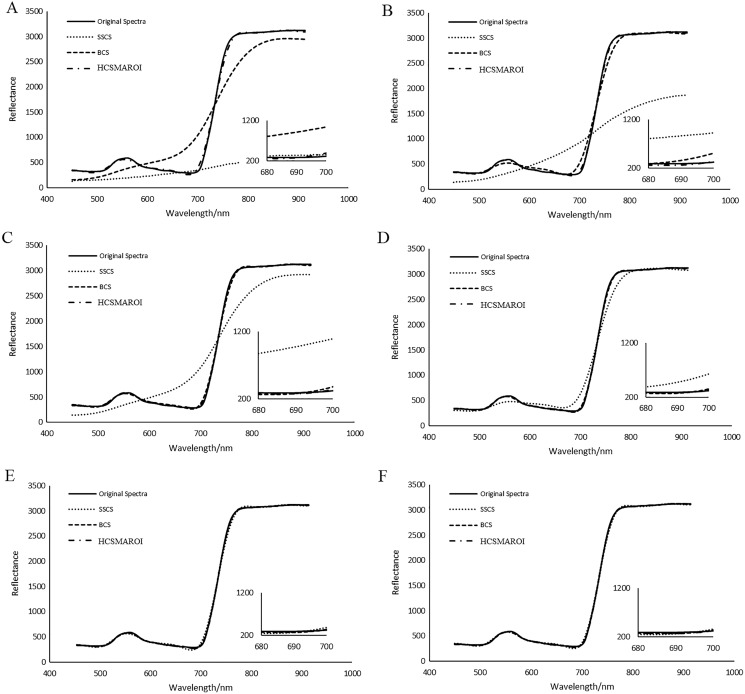
Comparisons of reconstructed spectra for the first tea leaf HSIs. (A) The whole sampling rate of 3%. (B) The whole sampling rate of 5%. (C) The whole sampling rate of 8%. (D) The whole sampling rate of 12%. (E) The whole sampling rate of 17%. (F) The whole sampling rate of 20%.

Spectral analysis of plant HSIs plays an important role in monitoring of plant growth, disease and insect pest detection and the inversion of physiological parameters. Figure 22 shows the average reconstructed spectra of the multiple arbitrary-shape ROIs of SSCS, BCS and HCSMAROI at different sampling rates from 5% to 20% for the first soybean leaf HSIs. Figure 23 shows that the average reconstructed spectra of the multiple arbitrary-shape ROIs of SSCS, BCS and HCSMAROI at the sampling rates from 3% to 20% for the first tea leaf HSIs. Experimental results shows that the reconstructed spectra of BCS are obviously closer to the original one than that of SSCS at different sampling rates. Besides, especially at low sampling rates, the reconstructed spectra of HCSMAROI are closer to the original ones than those of the others.

Table 4 gives RMSE comparisons of spectral indices of TVI and DD of the first soybean leaf HSIs for different algorithms at different sampling rates. For spectral indices of TVI and DD at different sampling rates, RMSE of HCSMAROI is relatively less than that of the other algorithms. At the sampling rate of 10%, HCSMAROI has especially obvious advantage. And RMSE of SSCS shows the largest RMSE values at different sampling rates.

**Table 4:** Comparisons of reconstructed RMSE of the spectral index TVI and DD for the first soybean leaf HSIs for different algorithms.

Spectral index	Algorithms	Sampling rates			
		5%	10%	15%	20%
TVI	SSCS	0.5369	0.0617	0.0289	0.0290
	BCS	0.0193	0.0135	0.0071	0.0034
	HCSMAROI	0.0151	0.0047	0.0013	0.0000
DD	SSCS	0.6605	0.1126	0.1476	0.1810
	BCS	0.1149	0.1596	0.1173	0.0909
	HCSMAROI	0.1635	0.0980	0.0680	0.0559

Table 5 shows RMSE comparisons of spectral indices of TVI and DD of tea leaf HSIs for different algorithms at different sampling rates. HCSMAROI can achieve obviously the best performance at low sampling rates of 3% and 5%.

**Table 5:** Comparisons of reconstructed RMSE of the spectral index TVI and DD for the first tea leaf HSIs for different algorithms.

Spectral index	Algorithms	Sampling rates					
		3%	5%	8%	12%	17%	20%
TVI	SSCS	0.9755	0.9172	0.6709	0.2066	0.0032	0.0060
	BCS	0.6236	0.1028	0.0096	0.0019	0.0005	0.0012
	HCSMAROI	0.0178	0.0015	0.0015	0.0023	0.0024	0.0021
DD	SSCS	0.9862	0.9646	0.8470	0.5427	0.2374	0.1618
	BCS	0.8329	0.4373	0.1740	0.0970	0.0671	0.0591
	HCSMAROI	0.2264	0.0999	0.0607	0.0452	0.0358	0.0320

### Reconstruction performance analysis for multiple arbitrary-shape ROIs using the labeled mask

In the “Reconstruction Performance Analysis of Multiple Arbitrary-shape ROIs using the Primary Mask”, the experimental results for multiple arbitrary-shape ROIs using the primary mask show that the proposed HCSMAROI can achieve significantly better reconstructed performance than that of the SSCS and BCS in the spatial and spectral domains. Therefore, HCSMAROI is chosen to perform the following experiments.

In this case, multiple arbitrary-shape ROIs can be compressed and reconstructed respectively using the labeled mask at different target sampling rates. This case can be regarded as a general one. Reconstruction of multiple arbitrary-shape ROIs using the primary mask is special situation of this case. For the first soybean leaf HSIs, the sampling rates of ROI1, ROI2, ROI3, and ROI4 are 5%, 10%, 15% and 20%, respectively. For the first tea leaf HSIs, the sampling rates of ROI1, ROI2, ROI3, ROI4, ROI5 and ROI6 are 3%, 5%, 8%, 12%, 17% and 20%, respectively. These ROIs can be compressed and reconstructed in turn according to their respective target rates. The whole sampling rate can be calculated as follow:

(9) $$R{\rm{a}}ti{o_{whole}} = {{\sum\limits_{{\rm{i}} = 1}^{\rm{n}} A re{a_{ROI({\rm{i}})}} \times p\left( i \right)} \over {Are{a_{whole}}}}$$

where $Are{a_{BOI\left( {\rm{i}} \right)}}$ and $p\left( i \right)$ are the area and sampling rate of the *ith* ROI respectively, $Are{a_{whole}}$ is the whole area of all ROIs in the primary mask, and $R{\rm{a}}ti{o_{whole}}$ is the whole sampling rate.

**Figure 24:**
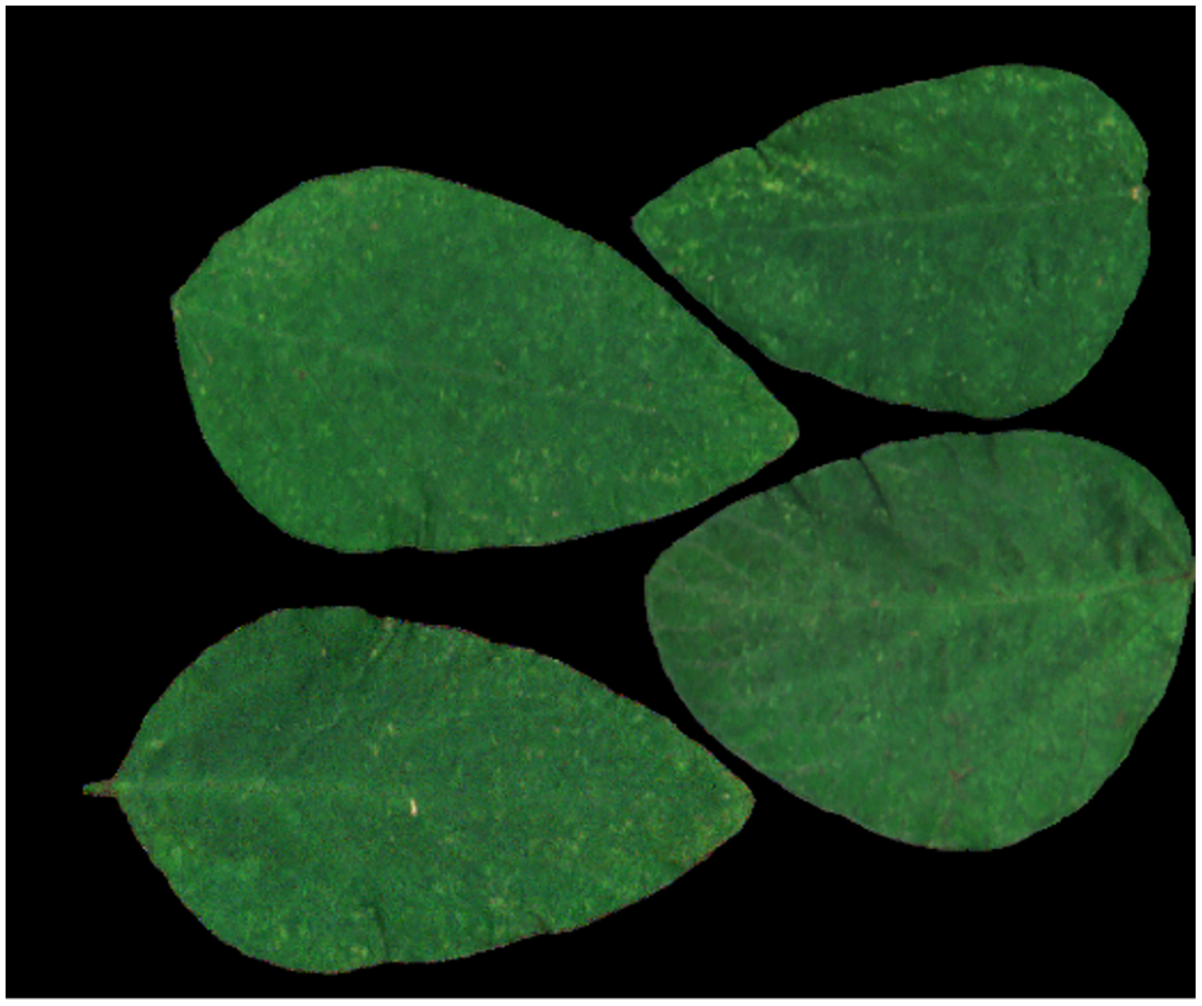
The reconstructed image for the first soybean leaf HSIs using the labeled mask.

**Figure 25:**
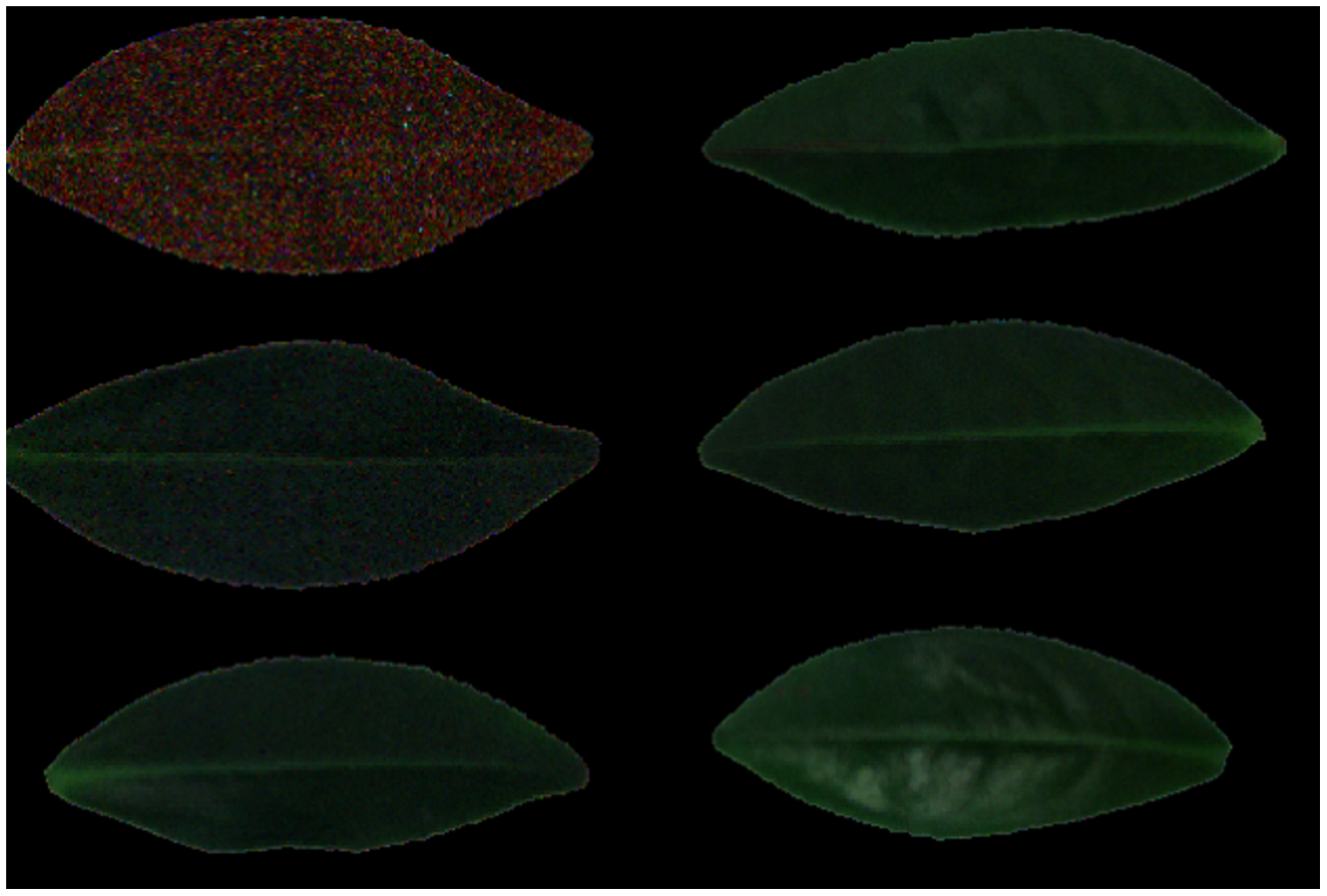
The reconstructed image for the first tea leaf HSIs using the labeled mask.

Figures 24 and 25 show the reconstructed images of the first soybean leaf HSIs and the first tea leaf HSIs, in which the ROI with higher sampling rate obtains better reconstructed quality, respectively. For soybean leaf HSIs, Table 6 also shows the same result that average reconstructed PSNR of ROI1 is the worst and that of ROI4 is the best. For tea leaf HSIs, Table 7 shows that average PSNR of reconstructed ROI1 is the worst and that of ROI6 is the best. These results shows that when the sampling rate of a certain ROI is greater than or equal to 10%, it can achieve quite good reconstructed quality in the spatial domain.

**Table 6:** Performance comparisons of reconstructed the first soybean leaf HSIs for different ROIs.

ROIs	ROI1	ROI2	ROI3	ROI4
Sampling rate	5%	10%	15%	20%
Average PSNR	33.12 dB	36.80 dB	38.24 dB	38.92 dB
TVI of reconstructed RMSE	0.0139	0.0135	0.0068	0.0047
DD of reconstructed RMSE	0.1162	0.2095	0.0752	0.1044

**Table 7:** Performance comparisons of reconstructed the first tea leaf HSIs for different ROIs.

ROIs	ROI1	ROI2	ROI3	ROI4	ROI5	ROI6
Sampling rates	3%	5%	8%	12%	17%	20%
Average PSNR	17.04 dB	27.71 dB	32.42 dB	33.60 dB	34.08 dB	34.11 dB
RMSE of TVI	0.6315	0.1056	0.0129	0.0006	0.0050	0.0087
RMSE of DD	0.8294	0.4330	0.1508	0.1011	0.0859	0.0671

**Figure 26:**
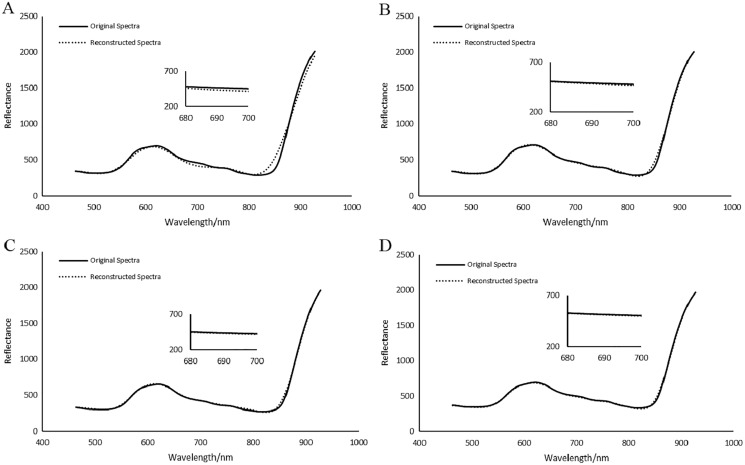
Comparisons of reconstructed spectra for the first soybean leaf HSIs for different ROIs. (A) The sampling rate of 5% for ROI1. (B) 2The sampling rate of 10% for ROI2. (C) The sampling rate of 15% for ROI3. (D) The sampling rate of 20% for ROI4.

**Figure 27:**
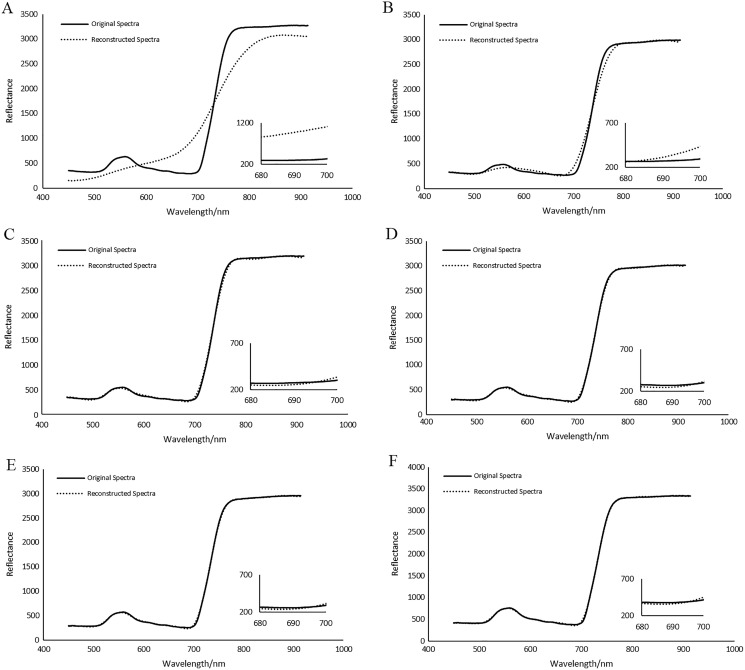
Comparisons of reconstructed spectra for the first tea leaf HSIs for different ROIs. (A) The sampling rate of 3% for ROI1. (B) The sampling rate of 5% for ROI2. (C) The sampling rate of 8% for ROI3. (D) The sampling rate of 12% for ROI4. (E) The sampling rate of 17% for ROI5. (F) The sampling rate of 20% for ROI6.

Figure 26 illustrates the reconstructed spectra of soybean leaf HSIs for different ROIs. For those ROIs whose sampling rates are larger than or equal to 10%, the reconstructed spectra can be quite closer to the original spectra. Figure 27 illustrates the reconstructed spectra of tea leaf HSIs for different ROIs. For those ROIs whose sampling rates are larger than or equal to 5%, the reconstructed spectra can be closer to the original spectra than the others. The higher the sampling rate of a ROI, the closer to the original spectra it is.

Table 6 and Table 7 show the RMSE of reconstructed spectral indices TVI and DD for the first soybean leaf HSIs and the first tea leaf HSIs, respectively. Similarly, the higher the sampling rate of a ROI, the smaller the reconstructed RMSE of reconstructed spectral indices TVI and DD. When the sampling rate of a certain ROI is greater than or equal to 15% for soybean leaf HSIs or 10% for tea leaf HSIs, the ROI can also achieve quite good reconstructed quality in the spectral domain.

Therefore, the proposed HCSMAROI can provide a flexible way to effectively compress and reconstruct different arbitrary-shape ROIs with different strategy meanwhile good reconstructed performance can be obtained in the spatial and spectral domains.

## Conclusion

There are high inter-spectral and inter-spatial correlations for plant leaf HSIs. The proposed HCSMAROI can achieve better reconstruction effects in both the spectral and spatial domains than those of SSCS and BCS. At low sampling rates, HCSMAROI shows quite good reconstructed performance. HCSMAROI can also be closer to the original spectra and retain the spectral indices of TVI and DD more effectively than those of SSCS and BCS. Besides, the proposed HCSMAROI shows special advantage that it can effectively compress and reconstruct different arbitrary-shapes ROIs with different sampling rates, and brings good reconstructed performance in both the spatial and spectral domains.

## Additional Information and Declarations

### Data Availability

The following information was supplied regarding data availability: The data is available at FigShare: Jia, Yuewei (2021): Raw data and codes.. figshare. Dataset. https://doi.org/10.6084/m9.figshare.16811236.v1.
